# Comparison of Inhibitory Capacities of 6-, 8- and 10-Gingerols/Shogaols on the Canonical NLRP3 Inflammasome-Mediated IL-1β Secretion

**DOI:** 10.3390/molecules23020466

**Published:** 2018-02-21

**Authors:** Su-Chen Ho, Yi-Huang Chang

**Affiliations:** Department of Food Science, Yuanpei University of Medical Technology, Hsinchu 300, Taiwan; yihuang@mail.ypu.edu.tw

**Keywords:** gingerols, shogaols, NLRP3 inflammasome, caspase-1, IL-1β

## Abstract

Endogenous noninfectious substances that mediate the nucleotide oligomerization domain (NOD)-like receptor family pyrin domain-containing 3 (NLRP3) inflammasome activation and interleukin (IL)-1β secretion causes inappropriate sterile inflammation and is implicated in the pathogenesis of several chronic diseases, such as type 2 diabetes mellitus, gout, atherosclerosis and Alzheimer’s disease. Consequently, dietary phytochemicals exhibiting capacities to suppress canonical NLRP3 inflammasome-mediated IL-1β secretion can be a reliable supplement to prevent such diseases. The purpose of this study was to investigate and compare the inhibitory effects of ginger phytochemicals, including 6-, 8- and 10-gingerols/shogaols on the canonical NLRP3 inflammasome-mediated IL-1β secretion in THP-1 macrophages with ordered stimulations of lipopolysaccharide (LPS) and adenosine 5′-triphosphate (ATP). At 20 μM, the 10-gingerol and all the shogaols significantly inhibited canonical IL-1β secretion. The shogaols had a more potent inhibitory capacity than that of corresponding gingerols. Increase of alkyl chain length impacted negatively the inhibitory activity of shogaols. Additionally, these effective ginger phytochemicals not only inhibited the LPS-primed expression of pro-IL-1β and NLRP3, but also decreased ATP-activated caspase-1. The results demonstrated that ginger phytochemicals, especially the most potent, 6-shogaol, might be promising for developing as an inhibitor of the canonical NLRP3 inflammasome-mediated IL-1β secretion and further applied in prevention of NLRP3 inflammasome-associated diseases.

## 1. Introduction

Inflammation is an essential innate immunological response of a host to infection and injury. However, long-term uncontrolled or inappropriate inflammation causes tissue damage, and eventually, results in disease occurrence. Interleukin (IL)-1β is a master proinflammatory cytokine that exerts systemic inflammatory or local immunological actions such as severing as a pyrogen to elevate temperature and enhance leucocyte migration, inducing the expression of acute phase proteins and adhesion molecules to recruit leucocytes as well as orchestrating the functions and differentiation of innate and adaptive lymphoid cells, and consequently, to affect all cells and organs [1,2,3,4]. The canonical secretion of IL-1β is rigorously controlled by a unique two-signal process. In response to the first stimulation of pathogen-associated molecular patterns (PAMPs), such as the lipopolysaccharide (LPS), macrophages are primed to trigger the intracellular NF-κB signaling cascade that further induces the expression of immature precursor of IL-1β (pro-IL-1β) and the nucleotide oligomerization domain (NOD)-like receptor family pyrin domain-containing 3 (NLRP3), a cytosolic pattern recognition receptor. Upon the second stimulation of PAMPs or damage-associated molecular patterns (DAMPs), NLRP3 recruits the adaptor molecules—apoptosis-associated speck-like containing CARD (ASC) and pro-caspase-1—to assemble an inflammasome complex; consequently, caspase-1 is activated and converts pro-IL-1β into mature IL-1β [5]. NLRP3 inflammasome responds to various endogenous DAMPs such as extracellular ATP, uric acid crystal, cholesterol crystal and β-amyloid plaque, and produces redundant IL-1β and brings so-called sterile inflammation. Such sterile inflammation is implicated in the pathogenesis of several noncommunicable diseases including type 2 diabetes, arthritis, gout, atherosclerosis, and Alzheimer’s disease. Hence, it has been reasonably recognized as an effective therapeutic strategy to prevent sterile inflammation associated diseases by blocking the canonical NLRP3 inflammasome-mediated IL-1β secretion [6,7,8]. Extracellular ATP released from necrotic cells triggers activation of NLRP3 inflammasome and IL-1β secretion via binding to the purinergic 2X7 (P2X7) receptor, causes sterile inflammation and contributes to the pathogenesis of ischemic diseases [9,10]. High concentrations of ATP (5 mM) strongly elicits the processing of pro-IL-1β into mature IL-1β in LPS-primed macrophages [11]. Consequently, the LPS-primed and ATP-activated THP-1 macrophage cell model has been widely adopted to evaluate the efficacy of the proposed IL-1β inhibitors [12,13,14]. 

**Figure 1:**
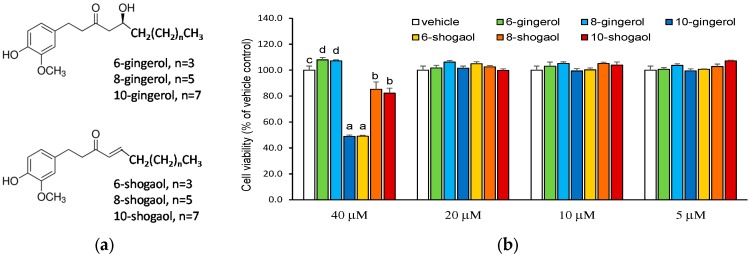
Chemical structure (**a**) and cytotoxic effect (**b**) of ginger phytochemicals. The phorbol 12-myristate 13-acetate (PMA)-differentiated THP-1 macrophages were treated with ginger phytochemicals at concentrations of 5~40 μM for 24 h. The cell viabilities were then assessed with 3-(4,5-dimethyldiazol-2-yl)-2,5-diphenyl tetrazolium bromide (MTT) method. Data are expressed as the mean ± SD of triplicate independent experiments. Values of the ginger phytochemicals at concentration of 40 μM with different superscript letters are significant different from one another (*p* < 0.05).

Gingerols and their dehydrated derivatives, shogaols, are identified as the main bioactive components in fresh and dried gingers, respectively. Gingerols with a thermally labile beta-hydroxy keto group that can readily undergo dehydration to form the corresponding shogaols with a common α,β-unsaturated carbonyl moiety (Figure 1a). The structure–activity relationship and the molecular mechanism underlying the anti-inflammatory capacity of gingerols/shogaols has been deeply explored by using the LPS-induced cell culture model [15,16,17,18], however, the information regarding their effect on NLRP3 inflammasome-mediated sterile inflammation is still obscure. Consequently, 6-, 8-, 10-gingerols/shogaols were selected to evaluate and compare their suppressing capacities on the canonical NLRP3 inflammasome-mediated IL-1β secretion in THP-1 macrophages by using LPS and ATP as first and second stimulations, respectively, to prime inflammatory nuclear factor (NF)-κB signaling pathway and to activate NLRP3 inflammasome. Additionally, in response to LPS, macrophages produce great amount of proinflammatory cytokines, including tumor necrosis factor (TNF)-α, IL-1β, and IL-6. The LPS-induced expression of proinflammatory cytokines is predominantly regulated at transcriptional level by NF-κB. TNF-α is another master proinflammatory cytokine that orchestrates the production of a proinflammatory cytokine cascade, affects inflammatory cell activation and recruitment, and plays a key role in the development of many chronic inflammatory diseases [19]. Thereby, TNF-α secretion and expression was used as a reference herein to observe the effect of ginger phytochemicals on LPS-priming step.

## 2. Results

### 2.1. Inhibitory Capacities of Ginger Phytochemicals on LPS-Primed and ATP-Activated IL-1β Secretion in THP-1 Macrophages

To avoid the cytotoxicity caused by the overload of tested ginger phytochemicals, the 3-(4,5-dimethyldiazol-2-yl)-2,5-diphenyl tetrazolium bromide (MTT) assay was first performed to determine the optimal treating concentrations of the ginger phytochemicals. As presented in Figure 1b, 10-gingerol and all shogaols at a concentration of 40 μM had a significant cytotoxicity compared with vehicle control. On the other hand, at 20 μM, all of the tested ginger phytochemicals did not cause cell death. Based on the above findings, the modulatory capacities of gingerols/shogaols on IL-1β secretion were evaluated at concentrations ≤ 20 μM.

**Figure 2:**
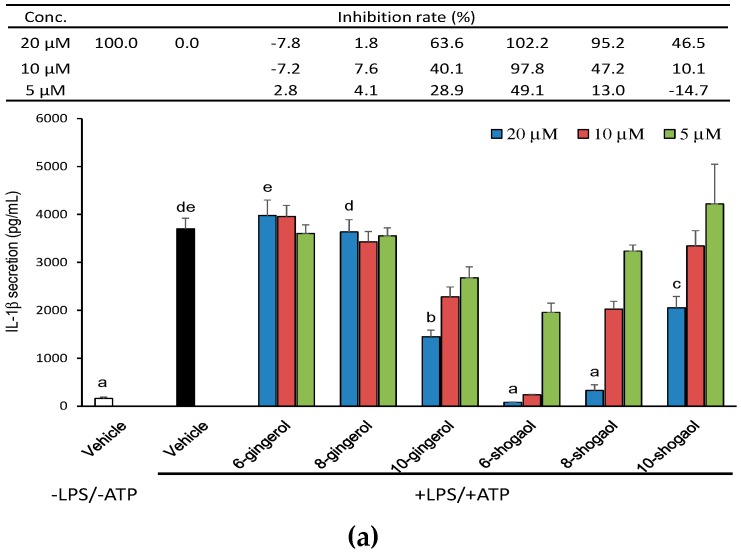

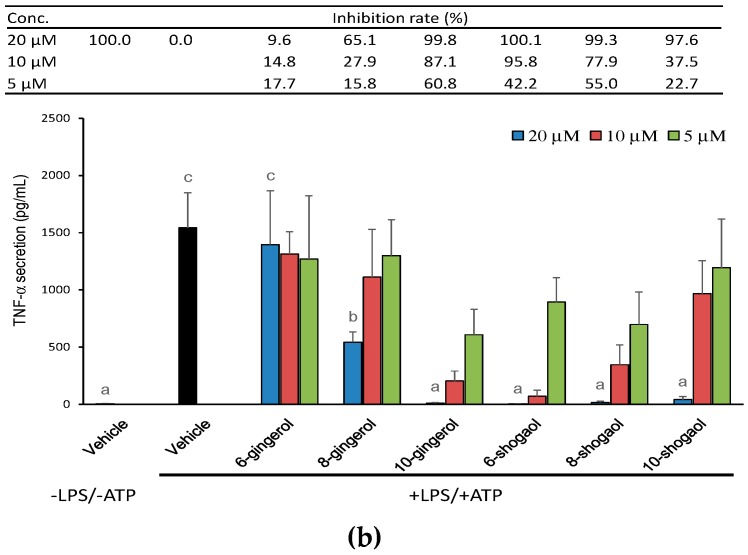
Effect of ginger phytochemicals on the LPS-primed and ATP-activated IL-1β (**a**) and TNF-α (**b**) secretion in THP-1 macrophages. The phorbol 12-myristate 13-acetate (PMA)-differentiated THP-1 macrophages were treated with ginger phytochemicals for 1 h. Then, the cells were primed with LPS for 4h and activated with 5 mM ATP for 3 h. The medium IL-1β and TNF-α concentration was assessed by an enzyme-linked immunosorbent assay (ELISA) kit. Data are expressed as the mean ± SD of triplicate independent experiments. Values of the ginger phytochemicals at concentration of 20 μM with different superscript letters are significant different from one another (*p* < 0.05).

In order to explore whether ginger phytochemicals inhibit the canonical NLRP3-mediated IL-1β secretion, THP-1 macrophages were treated with ginger phytochemicals before being LPS-primed and ATP-activated. Figure 2a shows the effect of pretreatment of ginger phytochemicals on IL-1β secretion in THP-1 macrophages. As expected, IL-1β secretion dramatically increased after LPS-priming for 4h and ATP-activation for 3h. All the tested ginger phytochemicals at concentrations of 5~20 μM, except 6-gingerol and 8-gingerol, inhibited LPS-primed and ATP-activated IL-1β secretion in a dose-dependent manner. Notably, 6-shogaol at concentrations of 10 and 20 μM as well as 8-shogaol at a concentration of 20 μM completely blocked IL-1β secretion. The inhibitory capacity of tested ginger phytochemicals was ranked as 6-shogaol (102.2%) > 8-shogaol (95.2%) > 10-gingerol (63.6%) > 10-shogaol (46.5%) in descending order from high to low. In addition, due to proinflammatory TNF-α expression/secretion primarily depends on the LPS-primed NF-κB signaling pathways. TNF-α secretion was used as a reference herein to observe the effect of ginger phytochemicals on LPS-priming step. Apparently, as shown in Figure 2b, except for 6-gingerol, the other ginger phytochemicals diminished dose-dependently TNF-α secretion. These results implied that ginger phytochemicals, especially 6-shogaol, exhibit potent ability to inhibit NLRP3 inflammasome-mediated IL-1β secretion and this inhibitory capacity might be attributed, at least partially, to their suppressing effect on the LPS-priming step.

### 2.2. Effect of Ginger Phytochemicals on Pro-IL-1β, NLRP3, Pro-Caspase-1 and Caspase-1 Protein Expression in LPS-Primed and ATP-Activated THP-1 Macrophages

**Figure 3:**
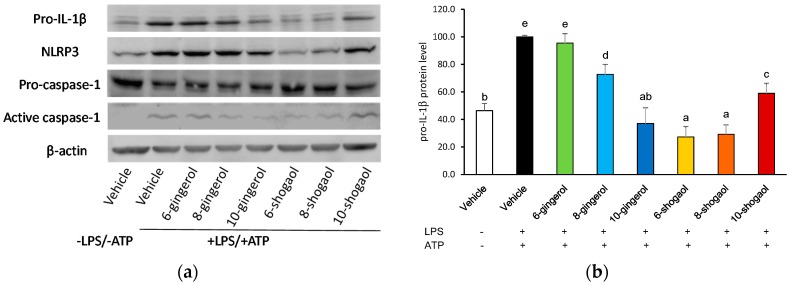

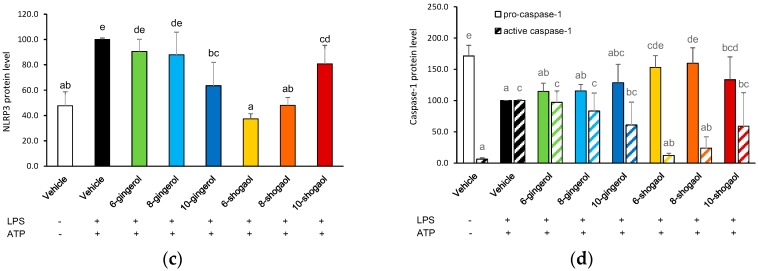
Effect of ginger phytochemicals on the protein levels of pro-IL-1β (**b**), NLRP3 (**c**), pro-caspase-1 (**d**) and active caspase-1 (**d**) in LPS-primed and ATP-activated THP-1 macrophages. The panels show a representative photogram of immunoblotting (**a**) and the quantitative results of pro-IL-1b (**b**), NLRP3 (**c**) as well as pro-caspase-1 and active caspase-1 (**d**), respectively. The PMA-differentiated THP-1 macrophages were treated with ginger phytochemicals (20 μM) for 1h, primed with LPS for 4h and then activated with 5 mM ATP for 3 h. Cytosolic proteins were subjected to determine protein levels of pro-IL-1β, NLRP3, pro-caspase-1 and active caspase-1 by immunoblotting. Data are expressed as the mean ± SD of triplicate independent experiments. Values with different superscript letters are significant different from one another (*p* < 0.05).

To investigate whether ginger phytochemicals affect the protein levels of pro-IL-1β, NLRP3, pro-caspase-1 and active caspase-1, cells were treated with ginger phytochemicals before being LPS-primed and ATP-activated. As shown in Figure 3, priming macrophages with LPS significantly enhanced the protein expressions of pro-IL-1β and NLRP3. At a concentration of 20 μM, the gingerols and shogaols, except for 6-gingerol, significantly decreased the LPS-primed pro-IL-1β protein level in THP-1 macrophages. The ability of these compounds to suppress pro-IL-1β protein expression was ranked as 6-shogaol (72.7%) and 8-shogaol (70.8%) ≥ 10-gingerol (62.9%) > 10-shogaol (41.0%) > 8-gingerol (27.2%) in the order from high to low (Figure 3b). Although the suppressing extent of ginger phytochemicals on the NLRP3 protein was weaker than those on the pro-IL-1β protein, the rankings of ability of these compounds to inhibit both proteins showed similar trend. On the other hand, the pro-caspase-1 level was decreased and the active caspase-1 level was elevated after LPS-priming and ATP activation. Pretreatment of 6-shogaol and 8-shogaol significantly attenuated the decrease of pro-caspase-1 and the elevation of active caspase-1 induced by LPS-priming and ATP activation (Figure 3d).

### 2.3. Effect of Ginger Phytochemicals on LPS-Primed mRNA Expression of IL-1β, NLRP3 and Inflammasome-Related Components in THP-1 Macrophages

**Figure 4:**
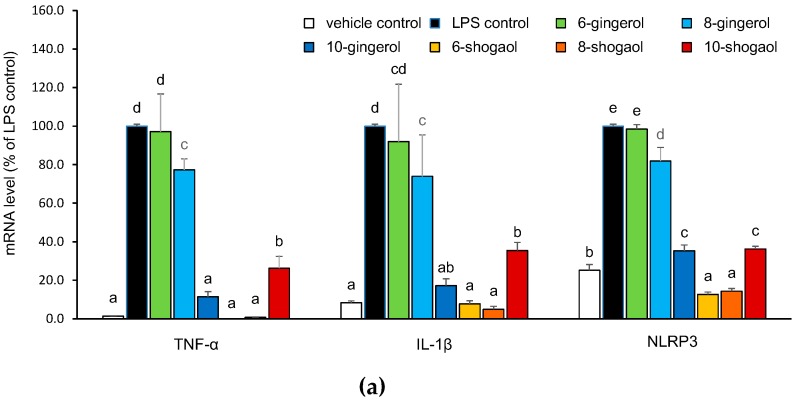

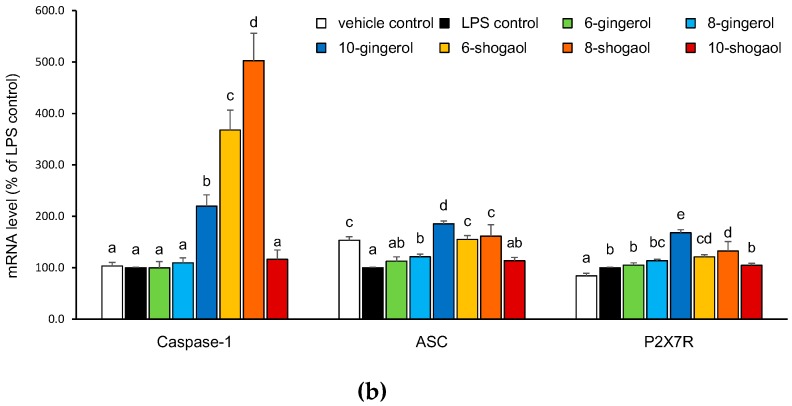
Effect of ginger phytochemicals on the mRNA expression of TNF-α (**a**), pro-IL-1β (**a**), NLRP3 (**a**), caspase-1 (**b**), ASC (**b**) and P2X7R (**b**) in LPS-primed THP-1 macrophages. The PMA-differentiated THP-1 macrophages were treated with ginger phytochemicals (20 μM) for 1h and primed with LPS for 3 h. Total cellular RNA was subjected to determine mRNA levels of target genes by quantitative PCR. Data are expressed as the mean ± SD of triplicate independent experiments. Values with different superscript letters are significant different from one another (*p* < 0.05).

Figure 4 shows the effect of ginger phytochemicals on the mRNA expression of TNF-α, IL-1β, NLRP3, caspase-1, ASC, and ATP-gated P2X7 receptor in LPS-primed THP-1 macrophages, respectively. According to Figure 4, LPS stimulation for 3h markedly increased TNF-α, IL-1β, and NLRP3, mildly enhanced P2X7 receptor. However, it did not influence caspase-1 and even decreased ASC mRNA expressions. Except for 6-gingerol, all other ginger phytochemicals attenuated LPS-primed TNF-α, IL-1β, and NLRP3 mRNA expressions. The effects of tested ginger phytochemicals on TNF-α, IL-1β, and NLRP3 mRNA show similar results and the suppressing ability was sorted as 6-shogaol and 8-shogaol ≥ 10-gingerol and 10-shogaol > 8-gingerol in progressive order from high to low. The results also indicate that the capability of the ginger phytochemicals to inhibit pro-IL-1β and NLRP3 expressions appear to be part of the molecular mechanism to explain the inhibitory capacity of these phytochemicals on the canonical NLRP3 inflammasome-mediated IL-1β secretion. In contrast, 6-shogaol, 8-shogaol and 10-gingerol seems to increase caspase-1, ASC and P2X7 receptor mRNA expression.

### 2.4. Inhibitory Capacities of Ginger Phytochemicals on ATP-Activated IL-1β Secretion in LPS-Primed THP-1 Macrophages

**Figure 5:**
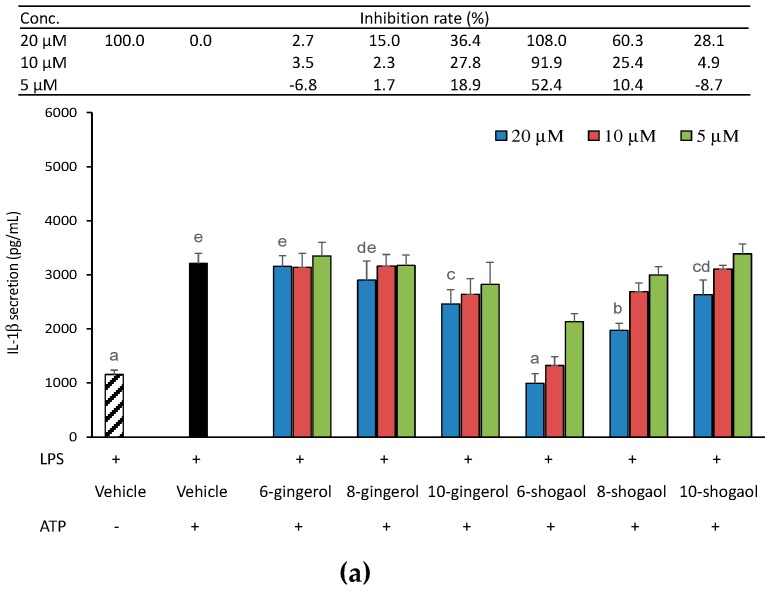

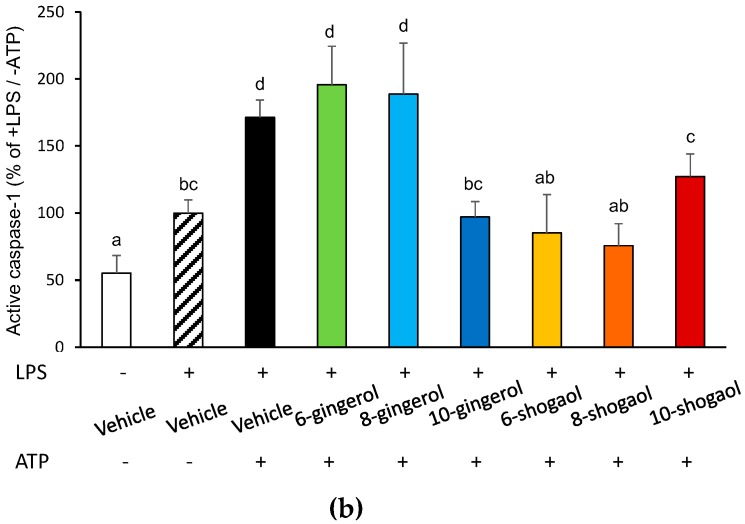
Effect of ginger phytochemicals on the ATP-activated IL-1β secretion (**a**) and caspase-1 activity (**b**) in LPS-primed THP-1 macrophages. The LPS-primed macrophages were treated with ginger phytochemicals for 1 h. Then, the cells were activated with 5 mM ATP for 3 h or 1 h, respectively, to determine IL-1β secretion and cytosolic active caspase-1. Data are expressed as the mean ± SD of triplicate independent experiments. Values of the ginger phytochemicals at concentration of 20 μM (**a**) and values (**b**) with different superscript letters are significant different from one another (*p* < 0.05).

To further explore whether ginger phytochemicals influence the NLRP3 inflammasome-mediated maturation process of IL-1β, macrophage cells were treated with ginger phytochemicals after being LPS-primed for 4 h and then activated with ATP for 3 h. The results are shown in Figure 5a. The LPS-primed THP-1 macrophages secreted 1157 and 3209 pg/mL IL-1β, respectively, under conditions of without or with further ATP (5 mM) activation. This result indicated that LPS stimulation not only affected priming step but also contributed partially to maturation process of IL-1β. On the other hand, the ATP stimulation boosted maturation process and obviously increased the secretion of IL-1β to 2.77-fold of that of LPS-primed alone. As presented in Figure 5a, clearly, at 20 μM, 10-gingerol and all tested shogaols effectively attenuated ATP-stimulated IL-1β secretion in LPS-primed cells. The concentration of the IL-1β secreted in the 6-shogaol treated cells was even equal to that secreted in the cells without ATP stimulation. At 20 μM, only 6-shogaol and 8-shogaol displayed a potent inhibitory ability (>50%) on ATP-activated maturation process of IL-1β.

Because active caspase-1 plays a key role in IL-1β maturation [20], it is essential to assess the effect of different ginger phytochemicals on the active caspase-1 levels. To address this issue, cellular active caspase-1 was determined with a covalently bound fluorescent inhibitor. As presented in Figure 5b, upon ATP activation, the cytosolic level of active caspase-1 was elevated to 171.5% of that of the LPS-primed cells. Also shown in Figure 5b, all the tested ginger phytochemicals at 20 μM, except 6-gingerol and 8-gingerol, demonstrated significant decrease of active caspase-1. 10-gingerol, 6-shogaol, and 8-shogaol completely diminished the activity of ATP-activated caspase-1. Inhibiting caspase-1 activation appeared to be one of the molecular mechanisms responsible for the suppressing capacity of these ginger phytochemicals on NLRP3 inflammasome-mediated IL-1β secretion.

## 3. Discussion

Gingerols/shogaols have been demonstrated to attenuate LPS-induced secretion of proinflammatory mediators through blocking the NF-κB-governed inflammatory signaling in macrophages, and thereby possess potent anti-inflammatory activity [16,17,21]. Moreover, 6-shogaol was documented to alleviate monosodium urate crystal-induced gouty arthritis and decrease the lysosomal enzymatic activity and the TNF-α level in mice [22]. It might be reasonably expected that gingerols/shogaols would have an inhibitory effect on the canonical NLRP3 inflammasome-mediated IL-1β secretion. Despite of this, to date, it is still unclear whether gingerols/shogaols could inhibit canonical NLRP3 inflammasome-mediated IL-1β secretion, whose structural feature might impact the inhibitory activity. Our results indicate, for the first time, that 10-gingerol and all the shogaols exerted significant inhibitory capacities on LPS-primed and ATP-activated IL-1β secretion at a concentration of 20 μM. The shogaols suppressed the canonical NLRP3 inflammasome-mediated IL-1β secretion more than the corresponding gingerols. This outcome implied that the inhibitory capacity of shogaols was primarily attributed to the presence of α,β-unsaturated carbonyl group in the chemical structures of these shogaols. Identically, the α,β-unsaturated carbonyl moiety was demonstrated to be the most critical determinant of the antioxidant, the anti-inflammatory and the antiproliferative potencies of shogaols [21,23,24]. Additionally, the increase of alkyl side chain length weakened the inhibitory capacity of shogaols on canonical NLRP3 inflammasome-mediated IL-1β secretion, while appearing to enhance those of gingerols. Correspondingly, the antineuroinflammatory, anti-invasive and antiproliferative activity of shogaols were also negatively impacted by the increase of the alkyl side chain length of shogaols [21,23,25].

The activation of transcription factor NF-κB, which induces proinflammatory genes expression, is recognized as the early event of canonical IL-1β secretion. In fact, NF-κB signaling is another predominant inflammatory cascade where the detailed molecular mechanism with regard to the inhibition effect of gingerols/shogaols on NF-κB governed inflammatory response has been well established. Gingerols/shogaols could interfere LPS-induced dimerization of toll-like receptor (TLR)-4, block PI3K/Akt, extracellular signal-regulated kinase 1/2 and p38 mitogen activated protein kinase (MAPK) signaling, inhibit NF-κB activation and translocation, and consequently, diminish LPS-induced expression of proinflammatory genes, such as iNOS, COX-2, IL-6, IL-1β and TNF-α [15,16,17,18]. In BV2 microglia, the suppressing ability of gingerols/shogaols on the LPS-triggered NF-κB activation was sorted as 6-shogaol and 8-shogaol > 10-gingerol and 10-shogaol > 8-gingerol. Simultaneously, the NF-κB suppressing abilities of gingerols/shogaols were parallel with their inhibitory extents on iNOS, IL-1β and TNF-α expressions [21]. In consistent with the previous study, a similar inhibitory trend of tested gingerols/shogaols on the LPS-primed expression of TNF-α, pro-IL-1β and NLRP3 was found herein. These results indicated inhibiting NF-κB governed proinflammatory genes expression at the priming step contribute to the inhibitory action of 10-gingerol/shogaols on the canonical NLRP3 inflammasome-mediated IL-1β secretion.

On the other hand, in contrast to NF-κB governed pro-IL-1β and NLRP3, the mRNA expression of caspase-1, ASC and P2X7 receptors were increased by 10-gingerol/shogaols. Lin et al. indicated that caspase-1 is expressed constitutively in THP-1 macrophages, and its mRNA and protein levels were not increased by LPS [26]. Based on the functional binding sites existing in the promoter region, the expression of caspase-1 gene is demonstrated to be activated by several transcriptional factors, such as IRF-1, signal transducer and activator of transcription (STAT) 1, p53, and p73 and E26 transformation-specific sequence 1 (Ets-1) [27]. On the other hand, 6-shogaol was demonstrated to induce lung cancer cell apoptosis through activating p53 pathway and to increase the expression of p21, p27, SOCS1, and IRF1 in prostate cancer cells [28,29]. The inducing effect of these effective ginger phytochemicals, especially 6-shogaol, on caspase-1 gene expression might be partially attributed to their activating effect on p53 and IRF1. However, this speculation needs to be proved in the future. Furthermore, the inducing molecular mechanism of 10-gingerol/shogaols on the ASC and P2X7 receptor mRNA expression is also unclear and should be investigated.

The second stimulation of extracellular ATP induce K^+^ efflux through an ATP-sensitive P2X7 receptor, to trigger assembly of NLRP3 inflammasome and, then, caspase-1 is activated by self-cleavage [7]. Our results showed that 10-gingerol, 6-shogaol, and 8-shogaol completely diminished the activity of ATP-activated caspase-1, however, only 6-shogaol completely decreased ATP-induced IL-1β secretion. In spite of this, it was demonstrated that inhibiting activation of caspase-1 also contributed to the inhibitory capacity of these ginger phytochemicals on the canonical NLRP3-mediated IL-1β secretion. Additionally, IL-1β processing enzyme other than caspase-1, such as caspase-8, might be regulated by ginger phytochemicals and resulted in increase of IL-1β secretion. This speculation needs to be proved.

Efflux of K^+^ and generation of reactive oxygen species (ROS) are two intracellular models to illustrate how the ATP triggers assembly of the NLRP3 inflammasome. [10,30]. However, due to the elevation effect of the shogaols and 10-gingerol on the mRNA level of P2X7 receptor in the LPS-primed cells, the blocking of efflux of K^+^ seems to be the least possible candidate for the mechanistic model to explain the caspase-1-inhibiting ability of these ginger phytochemicals. It leaves ROS reduction to be the more plausible model for the explanation of the inhibitory effect of these ginger phytochemicals. Gingerols/shogaols, particularly the 6-shogaol, possess substantial antioxidant ability and can scavenge various free radicals, such as superoxide and hydroxyl radical [24]. Additionally, 6-shogaol enhanced the enzymatic antioxidant defense system through the induction of nuclear factor erythroid 2 (NFE2)-related factor 2 (Nrf2) [31]. Peng et al., indicated that 6-shogaol conferred protection against oxidative stress-induced damage by both directly scavenging free radicals and activating endogenous cellular antioxidant defense system. They proposed that the phenoxyl group of 6-shogaol was responsible for directly counteracting against free radicals and the α,β-unsaturated carbonyl moiety contributed to activate the Kelch-like ECH-associated protein 1 (Keap1)-Nrf2-ARE signaling pathway by covalently modification of cysteine residues of Keap1. Oppositely, 6-gingerol lacking the α,β-unsaturated carbonyl moiety (Michael acceptor moiety) cannot modify Keap1 and thereby fails to shelter PC12 cells from oxidative stress [32]. Recently, Nrf2-mediated antioxidant signaling pathway has been documented to be a negative regulator of NLRP3 inflammasome through diminishing ROS production [33,34]. Based on the facts above, interference of NLRP3 inflammasome assembly by ROS reduction might contribute to the inhibitory activity of shogaols on the canonical NLRP3-inflammasome-mediated IL-1β secretion. Moreover, the inhibitory activity of shogaols might be partially attributed to their direct reversible or irreversible modification on cysteine residues of caspase-1 or NLRP3 ATPase [35,36].

## 4. Materials and Methods

### 4.1. Chemicals

LPS from *Escherichia coli* O55:B5, adenosine 5’-triphosphate (ATP), phorbol 12-myristate 13-acetate (PMA), dimethyl sulfoxide (DMSO), and 3-(4,5-dimethyldiazol-2-yl)-2,5-diphenyl tetrazolium bromide (MTT) for cell culture experiment were acquired from Sigma-Aldrich (St. Louis, MO, USA). The ginger phytochemicals, including 6-gingerol (C_17_H_26_O_4_), 8-gingerol (C_19_H_30_O_4_), 10-gingerol (C_21_H_34_O_4_), 6-shogaol (C_17_H_24_O_3_), 8-shogaol (C_19_H_28_O_3_), and 10-shogaol (C_21_H_32_O_3_) with a purity of >99% were procured from ChromaDex (Irvine, CA, USA). Cell culture medium and reagents were obtained from Thermo Fisher Scientific (Grand Island, NE, USA). All other chemicals used were analytical grade.

### 4.2. Cell Culture and Stimulation

Human THP-1 monocytes obtained from the Bioresource Collection and Research Center (Hsinchu, Taiwan) were cultured in RPMI 1640 medium supplemented with 2 mM l-glutamine, 25 mM HEPES, 10% fetal bovine serum, 100 μg/mL streptomycin, and 100 U/mL penicillin in a 37 °C fully humidified incubator with 5% CO_2_ atmosphere. THP-1 monocytes (1 × 10^6^ cells/mL) were seeded into 48-well plates, differentiated into macrophages by incubation with 100 nM PMA for 24 h, and starved further in fresh RPMI 1640 medium overnight. To evaluate the inhibitory effect of ginger phytochemicals on canonical IL-1β secretion, cells were treated with ginger phytochemicals (5~20 μM) for 1 h and then primed with 1 μg/mL LPS for 4 h and activated with 5 mM ATP for 3 h. Additionally, to evaluate the inhibitory effect of ginger phytochemicals on ATP-activated maturation process of IL-1β, LPS-primed cells were washed with fresh medium and then treated with ginger phytochemicals (5~20 μM) for 1 h and then activated with ATP for 3 h. The condition media were collected for IL-1β and TNF-α assay. The MTT assay was adopted to evaluate the cytotoxic effect of tested ginger phytochemicals on THP-1 macrophages. Briefly, the PMA-differentiated macrophages were treated with the ginger phytochemicals at concentrations from 5 to 40 μM for 24 h and then incubated with medium containing 0.5 mg/mL MTT for another 4 h. After which, the formed formazan was dissolved in DMSO and the absorbance at 550 nm was detected with a multimode reader (Infinite M200 PRO; Tecan Group Ltd., Männedorf, Switzerland).

### 4.3. Measurement of IL-1β and TNF-α Secretion

Medium IL-1β and TNF-α levels were determined by using an enzyme-linked immunosorbent assay (ELISA) according to the manufacturer’s recommendations (BioLegend, San Diego, CA, USA). 

### 4.4. Protein Extraction and Western Blotting

After the in-sequence treatment of ginger phytochemicals, LPS and ATP, THP-1 macrophages were collected and cytosolic proteins were isolated with a commercial kit (NE-PER nuclear and cytoplasmic extraction reagents, Thermo Fisher Scientific, Waltham, MA, USA). Target protein expressions were then determined with immunoblotting. Briefly, total cytosolic proteins were separated on a SDS-PAGE gel, transferred to a polyvinylidene difluoride (PVDF) membrane, and probed with anti-NLRP3 (NBP1-90207, Novus Biological, Littleton, CO, USA), anti-pro-caspase-1 (sc-56036, Santa Cruz Biotechnology, Dallas, TX, USA), anti-pro-IL-1β (ab2105, Abcam, Cambridge, MA, USA), or anti-β-actin (3598, Biovision, Milpitas, CA, USA) antibodies. Following washing, alkaline phosphatase-conjugated secondary antibodies were used. Immunoreactive protein bands were visualized by chemiluminescence (CDP-Start, PerkinElmer, Waltham, MA, USA) and the images were captured with a chemiluminescence imager (Fusion solo S; Vilber Co., Marne-la-Vallée, France). The densitometry of each target protein band was then quantified using Image J software (National Institutes of Health, Bethesda, MD, USA) and normalized with β-actin. 

### 4.5. RNA Extraction and Quantitative RT-PCR

Cells in six-well plates were harvested after treatment with 20 μM ginger phytochemicals for 1 h and followed by LPS priming for 3 h. The total RNA was extracted with Trizol reagent (Invitrogen, Carlsbad, CA, USA) and reverse-transcribed to cDNA using reverse transcription kit (Promega, Madison, WI, USA). The cDNA was then submitted to quantitative PCR on a StepOneTM real-time PCR system (Applied Biosystems, Foster City, CA, USA). Relative mRNA expression of target gene was calculated using the 2-ΔΔCT method with β-actin as reference gene [37]. The following primers were used: TNF-α-Fw: 5′-CAGAGGGAAGAGTTCCCCAG-3′; TNF-α-Rv: 5′-CCTTGGTCTGGTAGGAGACG-3′; NLRP3-Fw: 5′-CTACACACGACTGCGTCTCATCAA-3′; NLRP3-Rv: 5-GGGTCAAACAGCAACTCCATCTTA-3′; IL-1β -Fw: 5′-AAACAGATGAAGTGCTCCTTCCAGG-3′; IL-1β-Rv: 5′-TGGAGAACACCACTTGTTGCTCCA-3′; Caspase-1-Fw: 5′-GAATGTCAAGCTTTGCTCCCTAGA-3′; Caspase-1-Rv:5′-AAGACGTGTGCGGCTTGACT-3′; ASC-Fw: 5′-ATCCAGGCCCCTCCTCAGT-3′; ASC-Rv: 5′-GTTTGTGACCCTCCGCGATAAG-3′; P2X7 receptor-Fw: 5′- TGTGCCTACAGGTGCTACGCC-3′; P2X7 receptor-Rv: 5′-GCCCTTCACTCTTCGGAAACTC-3′; β-actin-Fw: 5′-GAGACCTTCAACACCCCAGCC-3′; β-actin-Rv: 5′-GGATCTTCATGAGGTAGTCAG-3′.

### 4.6. Measurement of Active Caspase-1

Active caspase-1 was measured with a covalently bound fluorescent inhibitor, FAM-YVAD-fmk (Immunochemistry Technologies, Bloomington, MN, USA). Briefly, the LPS-primed cells were sequentially treated with ginger phytochemicals and ATP for 1 h, FAM-YVAD-fmk were added and incubated for another 1 h. After which, cells were further incubated with fresh medium for 1 h to remove the unbound inhibitors. The fluorescence intensity was measured using a multimode reader with an excitation wavelength of 488 nm and an emission wavelength of 530 nm.

### 4.7. Statistical Analysis

Data are expressed as the mean ± SD of triplicate independent experiments. Statistical comparisons among different treatments were analyzed by the one way ANOVA followed by Duncan’s test. Differences were considered to be statistically significant for *p* values < 0.05. Statistical analyses were performed with the SPSS 22.0 software (IBM, Armonk, NY, USA).

## 5. Conclusions

Our results showed that 10-gingerol/shogaols, especially 6-shogaol, had a potent capacity to attenuate canonical NLRP3 inflammasome-mediated IL-1β secretion in THP-1 macrophages by using LPS and ATP as the priming and activation stimulation. They exerted dual inhibiting actions, targeting both the expression of pro-IL-1β and NLRP3 governed by NF-κB as well as the activation of caspase-1 triggered by assembly of NLRP3 inflammasome. Although it needs more efforts to explore the detailed molecular mechanism and its effectiveness in clinical studies, ginger phytochemicals, particularly 6-shogaol, have great potentials to play a vital role as a novel canonical IL-1β inhibitor and may be applied in the future to prevent sterile inflammation-associated diseases.
